# Monolithically 3D-Printed Microfluidics with Embedded µTesla Pump

**DOI:** 10.3390/mi14020237

**Published:** 2023-01-17

**Authors:** Kai Duan, Mohamad Orabi, Alexus Warchock, Zaynab Al-Akraa, Zeinab Ajami, Tae-Hwa Chun, Joe F. Lo

**Affiliations:** 1 Department of Mechanical Engineering, University of Michigan–Dearborn, Dearborn, MI 48128, USA; 2 Department of Internal Medicine, University of Michigan Medical School, Ann Arbor, MI 48109, USA

**Keywords:** Tesla turbine, microfluidics pumps, 3D printing

## Abstract

Microfluidics has earned a reputation for providing numerous transformative but disconnected devices and techniques. Active research seeks to address this challenge by integrating microfluidic components, including embedded miniature pumps. However, a significant portion of existing microfluidic integration relies on the time-consuming manual fabrication that introduces device variations. We put forward a framework for solving this disconnect by combining new pumping mechanics and 3D printing to demonstrate several novel, integrated and wirelessly driven microfluidics. First, we characterized the simplicity and performance of printed microfluidics with a minimum feature size of 100 µm. Next, we integrated a microtesla (µTesla) pump to provide non-pulsatile flow with reduced shear stress on beta cells cultured on-chip. Lastly, the integration of radio frequency (RF) device and a hobby-grade brushless motor completed a self-enclosed platform that can be remotely controlled without wires. Our study shows how new physics and 3D printing approaches not only provide better integration but also enable novel cell-based studies to advance microfluidic research.

## 1. Introduction

### 1.1. Benchtop Microfluidic Pumps

Lab-on-a-chip devices require a source of fluidic power. Conventional benchtop flow sources can be sorted into multiple types based on their driving force: gravity-driven pumps can output continuous flow, but their pressures vary over time. Flow from surface tension-driven pumps is also continuous with varying pressure. A recent study of gravity-driven and surface tension-driven flows by Sung et al. and Beebe et al. showed the disadvantages of these two pumps in long-time perfusion and flow control [1,2,3]. Precision syringe pumps can provide flow with minute pressure oscillations from a fixed syringe volume [4]. Pressure-driven pumps have accurate flow and pressure controls but are also limited by chamber volumes and unable to recirculate the flow [5]. Peristaltic pumps can siphon and recirculate flow, but their flows are highly pulsatile by nature [6,7]. Centrifugal flow à la rotating devices from Ren, Y. et al. can generate a wide range of flow rates [8]. However, centrifugal flow is directionally outward and cannot be recirculated back into the device. Overall, conventional pumps cannot combine continuous, non-pulsatile flow with flow recirculation for long-term microfluidic cell culture. In vivo, pulsatile flow is an inherent characteristic in large blood vessels and central to vascular homeostasis. For in vitro microfluidic models, however, pulsatile pressure waves create unwanted shear stress that can affect cell survival, differentiation, and biomechanical stimulation.

### 1.2. On-Chip Integrated Pumps

There are currently several novel techniques to achieve on-chip microfluidic pumps. These include acoustofluidic pumps, the technology used in inkjet print heads; electroosmotic pumps, which apply a voltage across a diffusion membrane to control ionic flow; electrolysis gas pressure pumps; capillary force siphoning à la paper microfluidics; thermal driven micropump that use thermal bubbles as pinching rollers to provide peristaltic flow; finger-actuated and solenoid driven pumps that employ deformable dome valves to displace fluid volumes; and integrated ion pumps designed for drug delivery application [9,10,11,12,13,14,15]. However, these integrated pumps cannot provide continuous, non-pulsatile flow in a recirculating manner for long-term cell culture. Thus, a microfluidic power source with recirculation function, continuous flow, and low shear stress is vital for the next-gen lab-on-chip devices. Boundary-layer pumps such as Tesla turbines can achieve these requirements in a recirculation form, and its miniaturization would solve our microfluidic pumping needs.

### 1.3. Tesla Turbine Miniature Pumps

Tesla turbines are characterized by a stack of concentric disks with predefined gaps and spiral fluid flow. The operation of a Tesla turbine relies on the continuous momentum transfer between the flow and the rotating disks within the fluid boundary layers. First patented by Nicola Tesla in 1913, Tesla turbines were initially applied to large-scale hydroelectric power generation [16,17], and subsequently became popular in miniaturized applications among research enthusiasts. Specifically, Couto et al., 2006, discussed the number of disks required for a Tesla turbine based on fluid mechanics [18]. Lemma et al., 2008, performed a numerical study to explore the viscous flow turbine and the results showed a 25% turbomachinery efficiency [19]. In recent years, Ensign et al. designed a multi-disc fan based on the Tesla turbine principle [20]. Moreover, computational modeling explored the design and optimizations of Tesla turbines. Ciappi et al.’s simulation results suggested that on a small scale and in miniature sizes, the Tesla turbine could be a valuable portable power generator [21]. Rusin et al. modeled a five-disk Tesla rotor and the simulation showed a doubling of efficiency compared to normal bladed turbines [22]. However, there was a lack of research on Tesla turbines at the microfluidic scale until our group’s efforts in 2016 [23]. In the previous study, we leveraged resin-based stereolithography 3D printing to achieve a miniature pump the size of a quarter. While that study demonstrated a µTesla pump-driven microfluidic gradient, we realized that the complicated fabrication and integration would hamper the adoption of µTesla technology.

### 1.4. 3D Printed Microfluidics

Microfluidics are microengineered devices that enable precise fluid flow and routing. In the past few years, 3D printing has expanded the microfabrication toolbox with non-planar, low-cost and faster fabrication processes. However, the resolution of consumer-grade 3D printing still lags behind that of traditional fabrication methods such as SU-8 molding.

Lithography-based 3D printing relies on a UV laser or other light source to polymerize photo-curable resin from a bath layer-by-layer. Consumer-grade machines with XY resolution down to 30 and 20 μm are available at prices from $500 to $2000, making them an affordable choice for fast microfabrication compared to the classical cleanroom processes [24]. Recent advances by Hua Gong et al. customized a 3D printer to print channels with dimensions of 18 μm × 20 μm [25]; a few microfluidic devices were fabricated for emulsion perfusion and droplet generation [26,27,28,29]. However, conventional resins leave unpolymerized components that are toxic to cells [30] and require an extra step to reduce the toxicity and avoid poly-dimethyl siloxane (PDMS) curing inhibition [31,32,33]. Researchers have also developed cell-compatible bio-resins for lithography-based 3D printing [34], but their print resolution, and especially cost efficiency, are far from optimal. Furthermore, studies have also demonstrated that residues from resin-printed molds prevent PDMS from curing itself, requiring extensive post-processing using vacuum ovens.

Fused deposition modeling (FDM) 3D printing is an alternative approach to fabricating integrated microfluidic devices. Consumer-grade FDM machines are more cost-effective, with starting prices around $300–$1500. In FDM printers, a stepper motor drives the extruder gears to push polymer filaments through a heated nozzle, which is moved across a heated print bed to deposit materials layer by layer [24,28,35]. Multiple cost-effective materials can be used, including polylactic acid (PLA), acrylonitrile butadiene styrene (ABS), polyethylene terephthalate (PET), polycarbonate, and their modified variations [36]. In contrast to resin-based 3D printing, FDM resolution depends mostly on the nozzle orifice size. Some research groups have printed microchannel molds as small as 100–1000 μm in width and 50–500 μm height [37]. The Nelson group improved the resolution by printing a microfluidic device with 100 μm transparent channels [38]. The biggest advantage in terms of efficiency is the short printing time (typically less than 1 h) and the lack of post-processing compared to resin-3D printing or traditional photolithography itself. The low-cost, flexible, and automated process of FDM 3D printing represents a powerful tool for microfluidic device fabrication.

**Figure 1:**
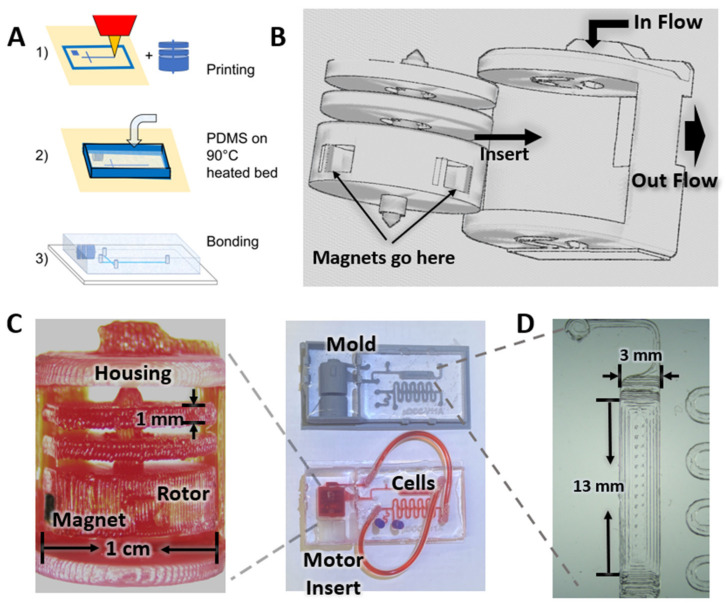
Microfluidic device fabrication method using 3D printing. (**A**) The microfluidics device mold was designed using AutoCAD and then printed using an FDM 3D printer. Microchannels and the µTesla pump were printed. The PDMS prepolymer is directly poured onto the heated bed and cured at 90 °C. The device is then bonded with a µTesla rotor inside. (**B**) Magnets were inserted in the µTesla rotor, the rotor was then assembled in the housing, flow comes from the side of the housing and the rotor rotation drives the flow pumping out of the µTesla pump. (**C**) PETG material was used to print the μTesla pump, with a rotor diameter of 1 cm. An integrated device was fabricated with μTesla pump assembled, with the main channel size being 700 μm in both width and height, and the smallest channel size being 200 μm in width and 700 μm in height. (**D**) Close view of the cell culture and differentiation chamber. The chamber size is 3 mm in width, 3 mm in height and 13 mm in length.

In contrast to most of the 3D microfluidics research, where enclosed channels are printed, we printed the negatives of the microfluidic geometry for standard PDMS soft-lithography molding. As a standalone technique, 3D printing has limitations in fabricating microfluidic devices due to the resolution, material, and biocompatibility issues. However, we leveraged the advantages of each technique—compatibility of PDMS molding and the robust automation of 3D printing—and created an integrated µTesla pump in a monolithic microfluidic system, as shown in Figure 1. The integration process was simple since most of the components, such as pump housing, siphon and output channels, were designed alongside the microfluidics and printed at the same time. The only manual step for integration was the insertion of the μTesla rotor into the device prior to plasma bonding. Due to the dimensions designed, the components slotted in with a snug fit that required no manual alignments. Our integrated pump empowered microfluidics research in long-term perfusion of cell culture, cell differentiation, and wireless operation of recirculating systems.

## 2. Materials and Methods

### 2.1. 3D Printing Optimization

The 3D prints were designed using Autodesk Inventor Professional 2023 (AutoDesk. Inc, San Rafael, CA, USA) and printed using a Voron Trident CoreXY 3D printer with polylite ASA filament (Polymaker, Houston, TX, USA). The slicing of .stl files into G-code for the Voron printer was completed using a CURA slicer (Ultimaker, Utrecht, The Netherlands) and the prints were optimized using nozzle diameters of 0.15 mm, 0.25 mm, and 0.4 mm. To print the patterns, the bed was heated to the desired temperatures such as 235 °C, 240 °C, and 245 °C before starting to print. To ensure bed adhesion, adhesive glue (Magigoo MPC2018, Thought3D, Paola, Malta) was added to the bed before printing. Two parameters were varied: temperature, and nozzle diameter. The printed patterns were 1 mm tall, with line widths/gaps of 100 µm, 200 µm, 300 µm, 400 µm, and 500 µm in dimension. To quantify the print quality, the channel, gap, and parts over gap ratios were calculated as follows:

(1) $$\mathrm{Channel}\;\mathrm{ratio}=\frac{\mathrm{Printed}\;\mathrm{line}\;\mathrm{width}}{\mathrm{Original}\;\mathrm{line}\;\mathrm{width}}\;$$

(2) $$\mathrm{Gap}\;\mathrm{ratio}=\frac{\mathrm{Printed}\;\mathrm{gap}\;\mathrm{width}}{\mathrm{Original}\;\mathrm{gap}\;\mathrm{width}}\;$$

(3) $$\mathrm{Parts}\;\mathrm{over}\;\mathrm{Gap}\;\left(\frac{P}{G}\right)=\frac{\mathrm{Printed}\;\mathrm{line}\;\mathrm{width}}{\mathrm{Printed}\;\mathrm{gap}\;\mathrm{width}}\;$$

The printed line width was the width of the line printed at three different temperatures with 3 different nozzle sizes. The same was defined for the printed gap widths. The original line and gap widths were the widths designed in AutoCAD 2022 (AutoDesk Inc., San Rafael, CA, USA), which are presented in Figure S1 as OSW and OGW, respectively.

### 2.2. 3D printing Roughness Measurement

A profilometer (Mitutoyo SJ-210, Sakado, Japan) was used to measure the average roughness, where the probe is used to detect the surface while physically moving to acquire the surface height. The surface roughness (*R_a_*) of the printed channels was measured along each channel width for 3 samples per mold. Profilometry demonstrated the surface roughness across a single line pattern versus nozzle sizes, temperatures, and designed channel width.

### 2.3. μTesla Pump Fabrication and Sterilization

The μTesla pump rotor and the pump housing were printed at 240 °C using a Voron filament 3D printer with a red PETG filament with a 0.25 mm nozzle. The rotor has a size of 1 cm, and the housing was designed to hold the rotor and allow it to rotate inside the housing. After printing, two magnets were inserted in the rotor in a polarized fashion. The rotor was then inserted into the printed pump housing to assemble the μTesla pump. The fabricated μTesla pump was soaked in 1 M hydrochloric acid (HCL) to increase positive surface charges and then washed with distilled water 5 times. After the HCL incubation, the pump was incubated in 1% sodium dodecyl sulfate (SDS) for 2 days for sterilization, and washed with PBS prior to device encapsulation [39].

### 2.4. Cell Culture Device Fabrication

The channels were printed in ASA at 240 °C using a 0.15 mm nozzle. The 3T3 L1 culture chamber size was 3 mm × 3 mm × 13 mm, the main channel size is 0.7 mm × 0.7 mm, and the smallest channel size is 0.2 mm × 0.7 mm, including droplet traps for future experiments, as shown in Figure 1B. A wall surrounding the microchannels was printed to allow PDMS molding. While the device was printing, PDMS prepolymer was prepared and degassed to save time. It was subsequently poured onto the 3D-printed mold directly on the print bed and baked at 90 °C for 1 h. The cured PDMS was then peeled from the print bed and bonded with a glass slide encapsulating a pre-printed μTesla rotor using plasma bonding. The bonded device was baked at 100 °C on the hot plate for 2 h before device sterilization for cell culturing.

### 2.5. Particle Velocimetry Analysis

The integrated microfluidic device was filled with PBS and operated at different rpm, from 1200 to 4000. The device inlet was connected to a tube of PBS solution and the outlet was connected to a 15 mL empty tube. By measuring the time for the pump to fill up the 15 mL outlet tube, we can drive the pumping flow rate at different rpm. To calculate the flow velocity, 10 μL fluorescent carboxylate-modified polystyrene latex beads (Sigma, Saint Louis, MO, USA) were dispersed in 2 mL distilled water; the beads have an average size of 1 μm. Diluted beads solution was injected into the integrated pump for the experiments. The pump was then run at 1500 rpm, which resulted in a flow rate around 50 μL/min. Fluorescent pictures were taken at 100 ms exposure time inside the cell differentiation chamber. The flow velocity was calculated by measuring the travel distance divided by the 100 ms exposure time.

### 2.6. 3T3 L1 On-Chip Culture and Differentiation

The 3T3 L1 cells were cultured in high glucose Dulbecco’s modified eagle medium (DMEM) (4500 mg/L glucose) with 10% bovine calf serum (BCS) inside a cell culture incubator. To culture the cell in the device, device channels were incubated with Poly-l-lysin solution (Fisher Scientific, Hampton, NH, USA) for half an hour to enable cell attachment [40]. 3T3 L1 cells at 1 × 10^7^ cells/mL concentration were loaded into the cell culture channel. The device was incubated for 5 h to allow 3T3 L1 cell attachment. During the experiment, the device was driven by a stir plate at 1500 rpm inside a cell incubator (Lab Water-jacketed Co2 Series Ii 3, Fisher Scientific, Hampton, NH, USA). After the cell density became over-confluent, the media was replaced with a differentiation media based on high glucose DMEM with 10% Fetal Bovine Serum (FBS), 1 μg/mL insulin, 0.5 mM methyl isobutylxanthine, and 0.25 μM dexamethasone (Fisher Scientific, Hampton, NH, USA). After 3 days of incubation in differentiation media, the media was replaced with high-glucose FBS media containing 1 μg/mL insulin for another 3 days [41]. All the cell experiments were operated with the μTesla pump rotating at 1500 rpm, resulting in a flow rate of around 50 μL/min.

### 2.7. Adipocytes Staining

The differentiated adipocytes were stained using an Oil Red O stain kit (Lipid (Oil Red O) Staining Kit, Sigma-Aldrich, St. Louis, MO, USA). According to the protocol, cells were fully differentiated, and the media was removed from microfluidic device channels and washed using PBS. 10% formalin was gently pumped into the channels and incubated for 30 min for cell fixation. After that, the formalin solution was discarded, and the cells were washed twice using distilled water. Cells were then incubated with 60% isopropanol for 5 min. After the isopropanol was removed, the Oil Red O working solution was added for 15 min. After this incubation, the cells were washed 5 times with distilled water and incubated with hematoxylin for 1 min. Finally, the cells were washed 5 more times with distilled water until no excess stain was seen. After staining, the device was imaged using a fluorescent microscope. Lipids droplets in adipocytes appeared red in color and the nuclei appeared blue in color [42].

### 2.8. Wireless µTesla Pump Operations

To achieve wireless operation, we designed an insert for a mini quadcopter brushless motor (2S, 0802 sizes, Happymodel, Quzhou, China) with a 10 mm neodymium donut magnet (N-S magnetized through the diameter, McMaster Carr, Elmhurst, IL, USA). By connecting this electric motor and magnet combination, we can drive the µTesla rotor using hobby-grade micro-RC (Wireless controller QAT-00001, Microsoft, Redmond, WA, USA) components. Furthermore, the motor/pump speed was monitored directly from the RC speed controller via a Bluetooth interface running on a cell phone (Flip3, Samsung, Suwon-si, Republic of Korea).

### 2.9. Statistical Analysis

All experiments were conducted at least in triplicates and repeated three times. The data represent the mean ± S.E.M. (standard error of mean) of three independent experiments. Statistical analysis was carried out using one-way analysis of variance (ANOVA) and linear regression analysis. The differences between the two sets of data were considered significant at *p*-value < 0.05.

## 3. Results and Discussion

### 3.1. Optimizing FDM 3D Printing for Integrated Microfluidics

**Figure 2:**
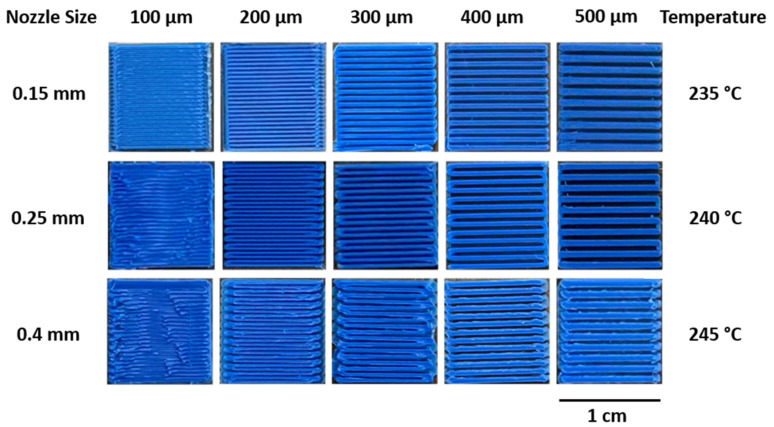
Printed patterns for FDM optimization. A pattern of lines and alternating gaps of equal widths were printed at 100, 200, 300, 400 and 500 µm widths. At their optimal temperatures, the smallest feature sizes were 100, 200 and 400 µm for 0.15, 0.25 and 0.4 mm nozzles, respectively.

We applied multiple line widths and gaps in 1 cm square patterns to optimize our FDM printing at various nozzle temperatures and orifice sizes, as shown in Figure 2. The use of these printed patterns is akin to the use of modulation transfer function in analyzing imaging optics, where spatial patterns better illustrate the imaging capabilities rather than single feature sizes. We note that our FDM optimization is not meant to compare with photolithography or resin 3D printing. However, we illustrate the capabilities of FDM printing as an integration technique for multiscale components.

**Figure 3:**
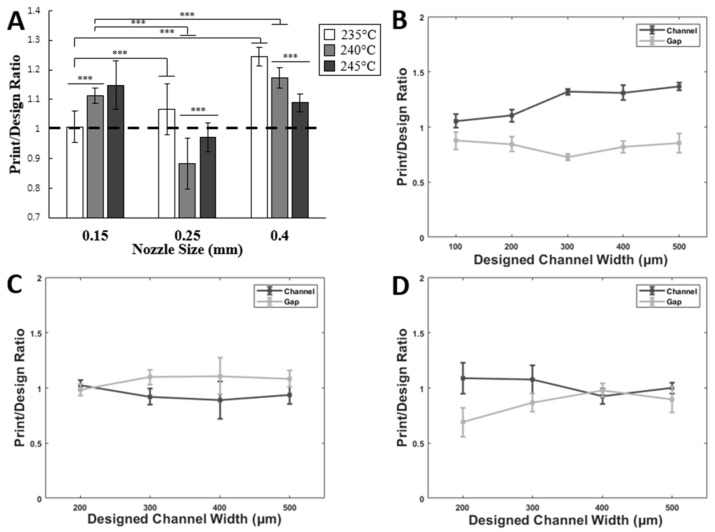
Optimizing FDM print resolution. (**A**) When ABS filaments were printed at various temperatures, accurate line widths compared to their nozzle sizes were obtained at 235, 245, and 245 °C for 0.15 mm, 0.25 mm, and 0.4 mm nozzles, respectively. At these optimal temperatures, alternating bands with line widths of 100 to 500 µm, were printed at equal spacings. Data are represented as a ratio of printed over designed parts. Error bar S.E.M (N = 3), *** *p*-value < 0.001. (**B**) For the 0.15 nozzle, the most accurate printed lines were obtained near the 100 µm designed width. Pearson’s *r* = 0.93 for channel and −0.17 for gap. (**C**) For the 0.25 nozzle, the most accurate printed lines were obtained near the 200 µm designed width. Pearson’s *r* = −0.83 for channel and 0.59 for gap. (**D**) For the 0.4 nozzle, the most accurate printed lines were obtained near the 400 µm designed width. These values allowed us to tailor slicer parameters that best suit the desired microfluidic geometries. Pearson’s *r* = −0.72 for channel and 0.78 for gap. Data are represented as a ratio of printed channel/gap over designed parts for the same channel/gap.

The ratio for the printed parts over designed parts was calculated for the three different temperatures and then the ratio for channels and gaps was found. Irrespective of the printing temperature, a 0.15 mm nozzle was shown to be the best size for printing channels below 200 µm, and a 0.25 mm nozzle for channels between 300 µm and 500 µm. As shown in Figure 3A, for 235 °C, the ratio was 1.03 ± 0.06 for the 0.15 mm nozzle, while the ratios for other nozzles of the same temperature were higher than 1, reaching 1.08 ± 0.15 for 0.25 mm and 1.26 ± 0.09 for the 0.4 mm nozzle (*p*-value < 0.001). However, a different trend was noticed for 240 °C and 245 °C where the ratios decreased from 1.12 ± 0.06 and 1.15 ± 0.05 for 0.15 mm nozzle into 0.88 ± 0.15 and 0.98 ±0.08 for 0.25 mm nozzle before it increased to reach 1.17 ± 0.04 and 1.09 ± 0.14 for 0.4 mm nozzle of 240 °C and 245 °C temperatures, respectively (Figure 3A) (*p*-value < 0.001). The temperature parameter showed significant improvements to the printed geometries with *p*-values of *** *p* < 0.001. Temperatures with optimal channel dimensions were employed for all subsequent protocols. *N* > = 3 for each condition tested, with *N* being number of repeat prints per condition.

To further investigate the printing ratios versus line width, printing over design ratios for channels and gaps were calculated. The ratios were found to be closer to 1 for the channel width of 100 µm and 200 µm of 0.15 mm nozzle, reaching 1.05 ± 0.06 and 0.87 ± 0.08 for 100 µm and 1.1 ± 0.05 and 0.84 ± 0.07 for 200 µm of channel and gap ratio, respectively (Figure 3B). The regression analysis for printing over design ratios showed a linear correlation between these values and initially designed strands for channels only with Pearson’s *r* of 0.93; however, the analysis for the ratios of the gaps showed a nonlinear correlation with Pearson’s *r* of −0.17, which means the error between the printed and the designed gaps is higher than that of the channels. The 100 µm designed channel width for the 0.25 mm nozzle was unprintable, as shown in Figure 2. However, the ratios for other channels and gap widths of the same nozzle were the best, with values slightly closer to 1, as shown in Figure 3C. The regression analysis for 0.25 mm showed a linear correlation for both channels and gaps, with Pearson’s *r* of −0.832 for channels and 0.59 for gaps. By contrast, a completely different trend was found for the 0.4 mm nozzle, with ratios close to 1 for channel measurements, and ratios not that far from the same value for the gap ones, as shown in Figure 3D. This can be attributed to over-extrusion while printing with high nozzle sizes and higher temperatures, resulting in gap values much lower than in the design. This was noticed in the regression analysis, where channels showed a completely linear correlation with Pearson’s *r* of −0.71. Statistical analysis showed that *p*-values < 0.001 for 0.4, 0.25, and 0.15 nozzles while comparing the three different temperatures. Linear regression showed that Pearson’s *r* was closer to 1 for all the channels and gaps apart from the gap ratios for 0.15 nozzle.

**Figure 4:**
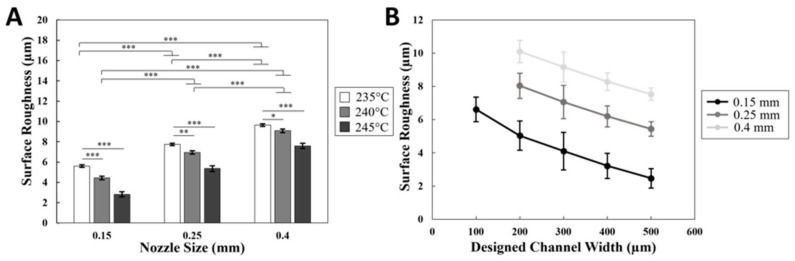
Surface roughness of printed channels. A Mitutoyo profilometer (Mitutoyo SJ-210, Japan) was used to characterize the surface roughness of the printed channels. (**A**) The roughness decreases with increasing nozzle temperatures and smaller orifice sizes. (**B**) Surface roughness decreases with increasing channel width and smaller orifice sizes. Error bar S.E.M (N = 3), * *p*-value < 0.05, ** *p*-value < 0.01, and *** *p*-value < 0.001.

To measure the average roughness for the channel surface of the molds printed, a profilometer (Mitutoyo SJ-210, Sakado, Japan) was used. Profilometry demonstrated the surface roughness across a single line pattern versus nozzle sizes, temperatures, and designed channel width. The data showed that the average surface roughness (*R_a_*) increased with increasing nozzle sizes and decreased with increasing temperatures, as shown in Figure 4A. The surface roughness decreased from 5.61 ± 0.12 for 235 °C to 4.43 ± 0.17 for 240 °C, and then to 2.82 ± 0.25 for 245 °C (*p*-values < 0.001). The same trend was found for the other 2 orifice sizes (0.25 mm and 0.4 mm). However, a different trend was shown when comparing the roughness with orifice sizes, as *R_a_* increased with the increase in size. For 235 °C, *R_a_* increased from 5.61 ± 0.12 for 0.15 mm, to 7.74 ± 0.12 for 0.25 mm, and then to 9.64 ± 0.12 for 0.4 mm (*p*-values < 0.001). The same trend was found for the other 2 temperatures. These results indicate that the roughness and orifice sizes are directly proportional, while roughness and temperatures are inversely proportional. These findings completely agree with the published data [43,44]. By contrast, *R_a_* decreased with increasing channel widths and also decreased for the smaller nozzle sizes, as shown in Figure 4B. Sample profilometer line scans are included in Figure S2.

### 3.2. 3D Printing Material Compatibility for Cell Culture

**Figure 5:**
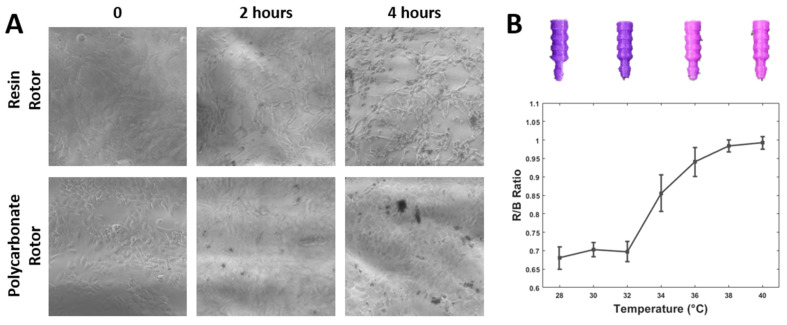
3D printing material comparisons. (**A**) Resin lithography and polycarbonate FDM-printed µTesla rotors were used in the devices to culture 3T3 L1 cells. Culturing with resin-printed rotors lead to cell detachment when compared to FDM-printed polycarbonate rotors after 4 h of incubation. FDM-printed rotors were adopted for subsequent cell culture. (**B**) The temperature of the plug changes from purple to pinkish when the temperature increases from 28 °C to 40 °C.

3D-printed microfluidic devices were coated with Poly-l-lysin to promote cell attachment. 3T3 L1 cells were loaded into the cell culture chamber and cultured in the incubator until confluency. Both resin-printed µTesla rotors and FDM-printed µTesla rotors were used to culture the cells in the device. Results showed that when resin-printed, µTesla rotors were used to pump the system, cell density decreased in 4 h, from 100% surface area to 30% surface coverage at the end of the 4 h pumping, as shown in Figure 5A.

### 3.3. Temperature-Sensitive Microfluidic Plugs

To enable monitoring of the cell culture outside of the incubator, plugs for fluidic ports were printed in temperature-sensitive PLA (37 °C blue to pink PLA). After cell loading, the loading port on the microfluidic was sealed with these color-changing plugs, providing visual monitoring of the temperature close to the cell culture chamber. The sensor calibration was carried out using image processing, whereby the ratio of red to blue intensities was plotted from 28 to 40 °C, as shown in Figure 5B. The calibration shows a sharp color change from 32 to 40 °C, allowing a quick assessment of cell culture temperature at a glance. These calibrated temperature plugs were used to monitor temperatures in our devices in a variety of settings. We used them within the cell culture incubator while changing media or incubating fluorescent dyes, and atop the microscope stage incubator.

### 3.4. Flow Rate and Particle Velocimetry Analysis

**Figure 6:**
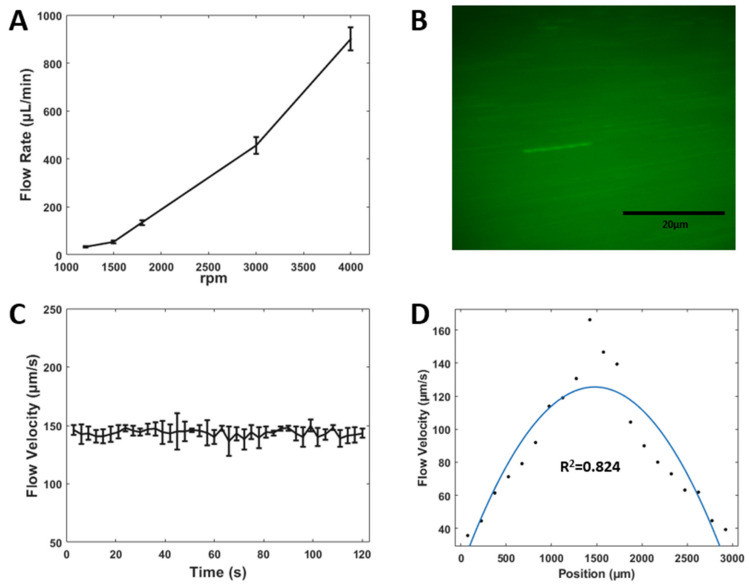
Flow rate and particle velocimetry for μTesla-driven microfluidics. (**A**) Flow rate increases with increasing μTesla rotation. Note that we operate cell culture perfusion at 1500 rpm or 50 μL/min. (**B**) Fluorescent microscopy of particles traveling in the center of the chamber. (**C**) Particle velocity was continuously measured at the center chamber with 100 ms exposure time at 3 s intervals for 2 min total. (**D**) Velocity profile inside the 3 mm wide cell culture chamber. A fitted parabola has an R^2^ value of 0.824, showing that the flow conforms to a laminar profile.

The µTesla pump, as a pressure pump, has different working conditions when applied to various microfluidic channel resistances. In our µTesla integrated microfluidics, increasing pump rotation from 1200 to 4000 increased the flow rate from 33 to 900 μL/min, as shown in Figure 6A. For cell culture experiments, we chose 1500 rpm or 50 μL/min for media perfusion. Particle velocimetry experiments were performed and measured in the cell culture chamber, as shown in Figure 6B. Velocity at the center of the chamber was sampled at 3 s intervals for 2 min, as shown in Figure 6C, showing a stable 150 μm/s velocity with a coefficient of variance of 4.67%. Across the 3 mm × 3 mm cross-section of the cell culture chamber, the velocity profile exhibited a parabolic shape characteristic of laminar flow, with an R^2^- value of 0.824, as shown in Figure 6D. The shear stress of the cell culture chamber was calculated when the pumps ran at 37 °C with a 50 μL/min flow rate. The results show the shear stress in the channel was around 0.6481 × 10^−3^ N/m^2^. These flow characteristics demonstrated that our μTesla integrated platform can provide a non-pulsatile and continuous laminar flow that is critical to cell culturing and precise microfluidic assays.

### 3.5. On-Chip Cell Culturing and Differentiation

**Figure 7:**
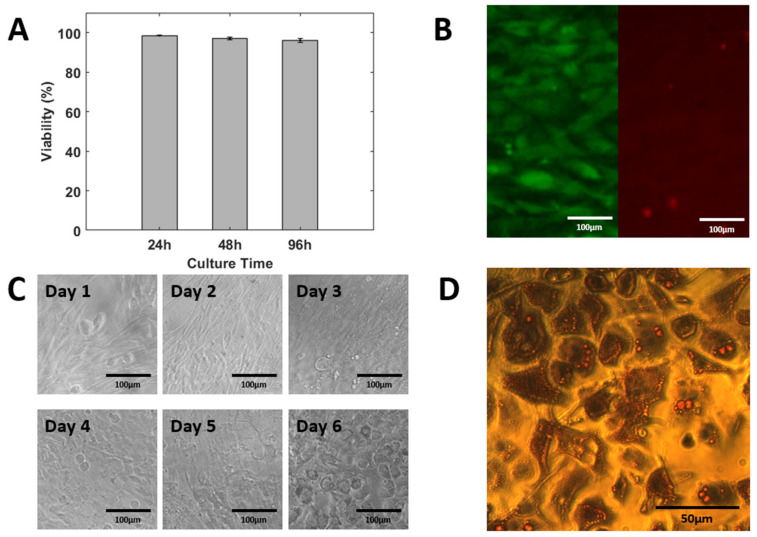
On-chip cell culturing and differentiation of 3T3 L1 cells to adipocytes. (**A**) Viability was assayed up to 96 h of culturing. (**B**) Live dead assay after 96 h culture. (**C**) On-chip differentiation can be seen up to Day 6. (**D**) Fat droplets were stained red and visualized under the microscope. This demonstrated the ability of our integrated pump to maintain long-term on-chip cell culturing.

We fabricated the cell culture device with an integrated Polyethylene terephthalate glycol (PETG) printed µTesla pump. The device included a rotor housing, motor housing, and a cell differentiation chamber to achieve long-term culturing. Poly-l-lysin coated device was used to culture cells for 24, 48, and 96 h, with a sterilized PETG µTesla pump providing medium recirculation. The results showed that cells achieved a greater than 98% survival rate after 24 h of culturing, and 95% of the cells were alive after 96 h of culture, as shown in Figure 7A,B.

3T3 L1 cells were loaded in the device and incubated until over-confluency, followed by incubation in differentiation medium for 3 days, insulin medium for 3 days, and finally FBS medium for 2 days, as shown in Figure 7C. The cells were stained with Oil Red O dye. Lipid droplets in the cells were stained red while the cell nucleus were stained blue. The differentiated cells have high counts of red droplets in their cytosols. The on-chip differentiation yielded more than an 80% differentiation ratio, as shown in Figure 7D. By leveraging the µTesla pump to power the microfluidic device, we achieved long-term cell culturing and differentiation. This represents a powerful tool for future cell-based lab-on-a-chip applications.

We want to emphasize the advantage of our μTesla pump in providing integrated recirculating flow that allowed for such a long-term cell culture on the chip. Without continuous perfusion, the 3T3 cells cannot survive beyond the differentiation process on a chip. In addition, pPetri-dish-differentiated 3T3 cells cannot be harvested and reseeded without losing differentiation. Achieving this length of culturing allows new disease models such as the adipose-beta co-culturing models of diabetes to be possible.

### 3.6. Wireless Operations

**Figure 8:**
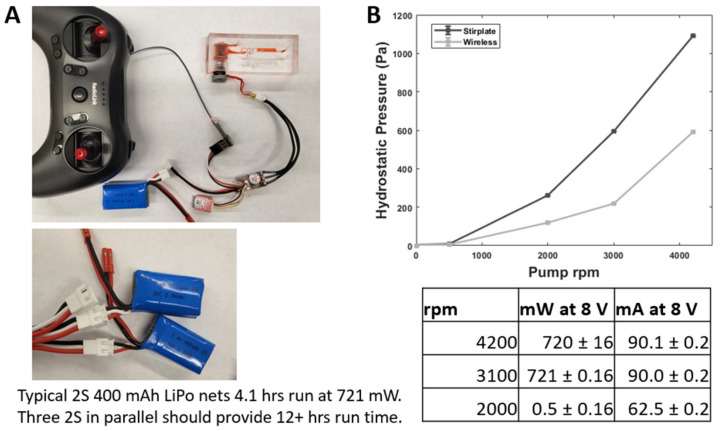
Wireless operation of Integrated µTesla Pump. (**A**) Wireless operation was enabled by inserting a miniature quadcopter brushless motor into the device and powering it using a micro speed controller and RF receiver. The speed was controlled by the RF joystick (on the transmitter) and monitored via a Bluetooth app on a smartphone not pictured. (**B**) The pump output under wireless operation was lower than the output when driven by a laboratory stir plate. The difference was attributed to brushless motor cogging below its designed speeds, which prevented steady µTesla rotations. The table summarized the power consumption of the wireless operation of the µTesla pump. At a maximum of 4000 rpm, the power draw was 721 mW. With multiple LiPo batteries in parallel, the run time can be extended indefinitely.

To demonstrate the utility of our 3D-printed microfluidic platform, we compared two types of device operations: (1) benchtop stir plate, and (2) miniaturized RC modes. Upon PDMS bonding, the encapsulated rotor with magnets can be directly actuated using a standard laboratory stir plate, in the same manner as our µTesla 1 [23]. In addition, we can wirelessly actuate the rotor using a micro brushless motor with magnetic coupling, as shown in Figure 8A. There were minimal differences between the stir plate and wireless operations in terms of output pressures, as shown in Figure 8B. At a max speed of 4200 rpm, the power draw at 8 V or full voltage for the 2S battery was 720 mW, as shown in Figure 8. When used with a 2S 400 mAh LiPo battery (2S 7.4V 30C, Crazepony, Shenzhen, China), the entire system ran for 4 h without fully depleting the battery. Connecting multiple 2S LiPo batteries in parallel increased the runtime and allowed for quick swapping for indefinite operation in cell culture applications.

## 4. Conclusions

In our view, FDM-printed microfluidics are not meant to compete in terms of resolution with classic SU8 photolithography, nor are they meant to achieve the low cost of stamped and roll-to-roll manufacturing for industrial-scale microfluidics. What the current evolution of FDM microfluidic excels at is the ability to span the micro to millimeter-scaled dimensions, and do so in a reliable, automated fashion. For applications that do not require channel resolution smaller than 100 µm, such as the 3T3L1 cell culture device, any novice student can learn to operate the printer to fabricate the entire PDMS in 2 h. Furthermore, 3D printing in general, including FDM and higher-resolution resin printing, enables novel three-dimensional structures such as overhanging channels and non-planar cylinders and cones to be fabricated easily. The one multi-material print we demonstrated here was a clever temperature-sensitive plug for the quick visual monitoring of cell culture temperatures. However, numerous novel materials are already available, including conductive filaments, ferromagnetic filaments, and water-soluble filaments, with more being developed at a rapid pace. The most important contribution of this work was the development of an integrated microfluidic pump, with its non-pulsatile and recirculating flow. Furthermore, we achieved this fabrication not through complicated microfabrication, but by using a low-cost and fully automated 3D printing technique (stl files are presented in S3 differentiation microfluidic mode.stl, S4 Pump housing.stl and S5 Pump rotor.stl). There are numbers of complex microfluidics that can be accelerated and optimized using our integrated prototyping, for instance digital microfluidics [45], disposable nucleic assay amplification tests [46] and co-culture disease models [47]. For these and future bioengineering endeavors, we are proposing an integrated electromechanical microfluidic fabrication that is available at the push of a “print” button.

## Data Availability

Not applicable.

## Supplementary Materials

The following supporting information can be downloaded at: https://www.mdpi.com/article/10.3390/mi14020237/s1, Figure S1: 3D printed models and design, Figure S2: surface roughness. S3: s3. differentiation microfluidic mode.stl, S4: s4. Pump housing.stl, S5: s5. Pump rotor.stl.
