# Supplementary material for: Monolithically 3D-Printed Microfluidics with Embedded µTesla Pump
Source: Micromachines (Basel). 2023 Jan 17;14(2):237. doi: 10.3390/mi14020237 (PMC9965163; doi:10.3390/mi14020237)
Supplement: Supplementary file 1 [file micromachines-14-00237-s001.zip › micromachines-2107337-Supplementary .pdf]

Supporting Information

Kai Duan [1], Mohamad Orabi [1], Alexus Warchock [1], Zaynab Al-Akraa [1], Zeinab Ajami [1], Tae-Hwa Chun [2] and Joe F. Lo [1,*]

1. Department of Mechanical Engineering, University of Michigan–Dearborn, Dearborn, MI 48128, USA
2. Department of Internal Medicine, University of Michigan Medical School, Ann Arbor, MI 48109, USA

A Voron Trident FDM 3D printer (Polymaker, Houston, USA) was used to print the microfluidics channel molds. To optimize the printing resolution, first, we used Inventor to design channels with different widths. Then we used nozzles with different sizes (0.15 mm, 0.25 mm, and 0.4 mm) to print the channels at different temperatures (235 °C – 245 °C). The prints have an overall size of 1 cm × 1 cm with 100 µm, 200 µm, 300 µm, 400 µm, and 500 µm line and gap widths.

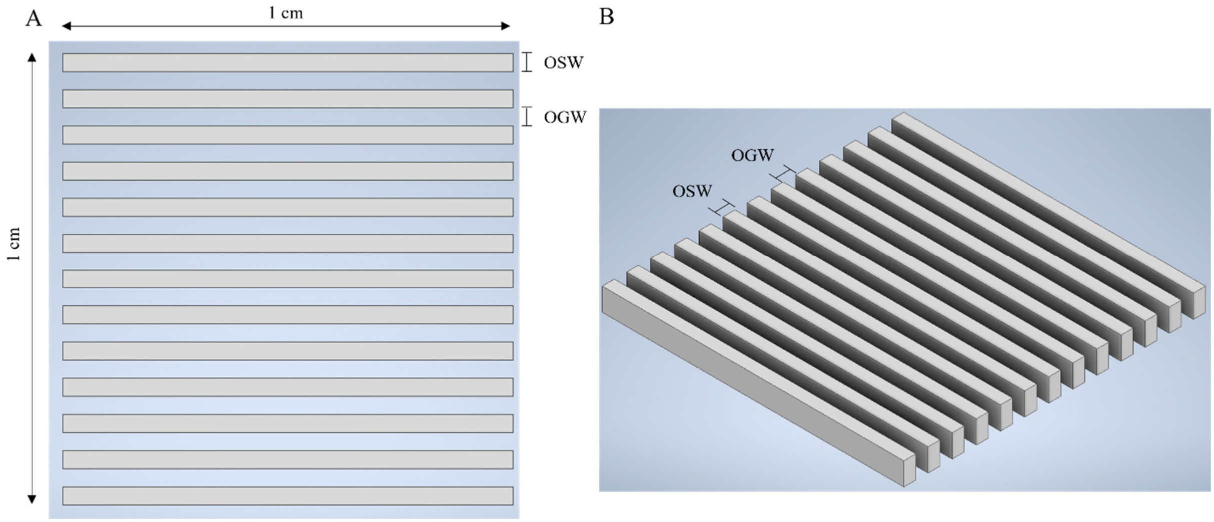

Supplementary Figure S1. 3D printed models and design. (**A**) Top view and (**B**) side view of the 3D print.

A profilometer (Mitutoyo SJ-210, Sakado, Japan) was used to measure the average horizontal roughness where the probe is used to detect the surface while physically moving to acquire the surface height. The surface roughness (R_a) of the printed channels was measured along each channel width for 3 samples per mold. An example of the roughness testing experiments is shown below for a 0.15 mm nozzle at 245 °C for a channel width of 100 µm, 300 µm, and 500 µm. The trend is obviously shown where the roughness is decreased with the increase in channel width.

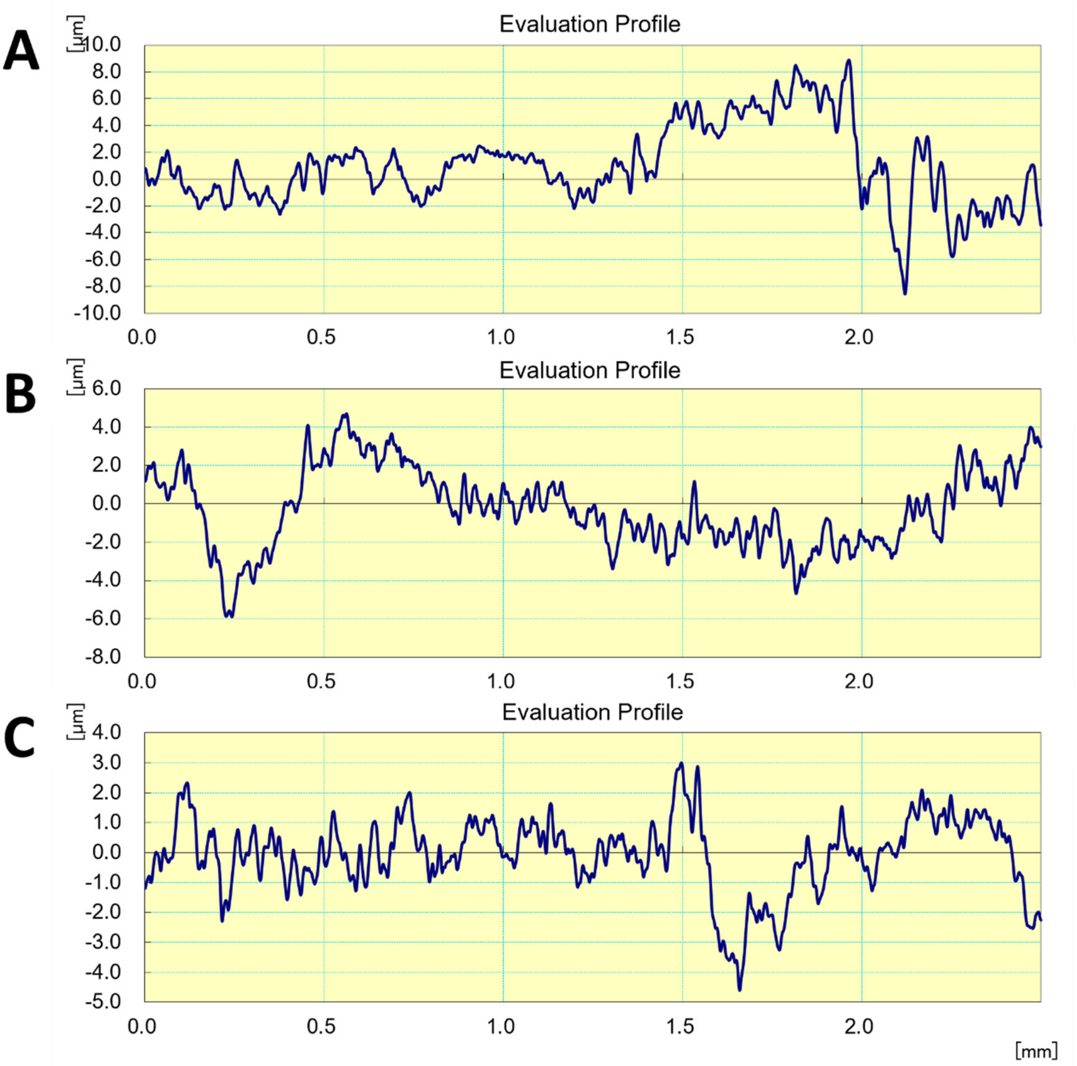

Supplementary Figure S2. The average roughness for 0.15 mm nozzle at 245 °C for (**A**) 100 μm, (**B**) 300 μm, and (**C**) 500 μm channels.
